# Analogue encoding of physicochemical properties of proteins in their cognate messenger RNAs

**DOI:** 10.1038/ncomms3784

**Published:** 2013-11-20

**Authors:** Anton A. Polyansky, Mario Hlevnjak, Bojan Zagrovic

**Affiliations:** 1 Department of Structural and Computational Biology, Max F. Perutz Laboratories, University of Vienna, Campus Vienna Biocenter 5, A-1030 Vienna, Austria

## Abstract

Being related by the genetic code, mRNAs and their cognate proteins exhibit mutually interdependent compositions, which implies the possibility of a direct connection between their general physicochemical properties. Here we probe the general potential of the cell to encode information about proteins in the average characteristics of their cognate mRNAs and decode it in a ribosome-independent manner. We show that average protein hydrophobicity, calculated from either sequences or 3D structures, can be encoded in an analogue fashion by many different average mRNA sequence properties with the only constraint being that pyrimidine and purine bases be clearly distinguishable on average. Moreover, average characteristics of mRNA sequences enable discrimination between cytosolic and membrane proteins even in the absence of topogenic signal-based mechanisms. Our results suggest that protein and mRNA localization may be partly determined by basic physicochemical rationales and interdependencies between the two biomolecules.

## Abstract (web summary)

mRNA transport contributes to the proper localization of its cognate proteins. Here the authors report a correlation between the physicochemical properties of mRNAs and their cognate proteins, suggesting that these properties of mRNAs can predict the subcellular localization of their cognate proteins.

## Graphical abstract

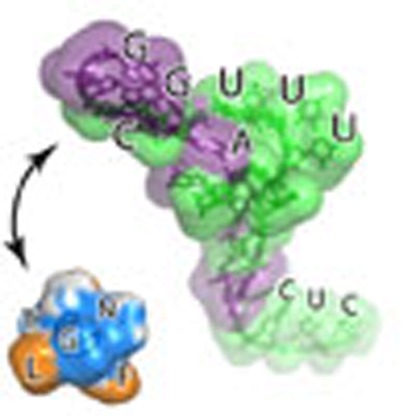

Accurate localization of proteins to a particular subcellular site, compartment or organelle represents one of the most important aspects of functioning of the living cell. Mechanisms ranging from those mediated by the specific action of cellular transport machinery^1^,^2^ to those based on random or biased diffusion^3^,^4^,^5^,^6^,^7^ have all been utilized to rationalize how proper cellular localization of proteins is achieved on different time- and length-scales. Moreover, a number of recent studies have revealed a major contribution of messenger RNA (mRNA) transport to the proper localization of their cognate proteins^8^,^9^,^10^,^11^,^12^,^13^,^14^,^15^,^16^. In general, most known cellular schemes for encoding the target locale of either proteins or mRNAs typically involve short, specific *cis*-acting motifs such as signal peptides for protein targeting or mRNA-embedded zipcodes for mRNA trafficking, which are in turn recognized by various *trans*-acting factors. The latter include signal-recognition particle, chaperones and RNA-binding proteins, but also different cytoskeletal elements and molecular motors of the cellular transport machinery^16^,^17^.

The signal-recognition particle-based mechanism, in particular, is responsible for directing secretory and membrane proteins to the prokaryotic plasma membrane or eukaryotic endoplasmic reticulum (ER) and it directly depends on ribosomal translation of N-terminal signal sequences. However, translation-independent localization of a number of mRNAs to ER has recently been discovered, including those transcripts that code for endomembrane proteins^12^,^13^,^18^. What is more, ribosome-independent membrane localization of bacterial mRNAs coding for membrane proteins has also recently been described^19^. In particular, it was demonstrated that segments of a membrane protein’s mRNA, which exclusively code for its soluble domains, remarkably resided in the cytoplasm, while those coding for its transmembrane domains associated with the membrane. Finally, although zipcode-based mechanisms have been shown to be important for accurate mRNA targeting, computational search for clear localization signals in mRNA sequences has been surprisingly difficult^20^,^21^. One still largely unexplored possibility is that the information actually resides in the more diffuse, general physicochemical properties of mRNA subsequences themselves.

Considering that mRNAs and cognate proteins are polymers with mutually interdependent composition as determined by the genetic code, here we examine the possibility that an mRNA transcript could encode and present to the cell information about the physicochemical characteristics of its product without being read and deciphered on the ribosome. For example, is it possible for the hydrophobicity of a protein, which is directly related to its final cellular destination, to be in an analogue fashion encoded in the hydrophobicity of its cognate mRNA sequence, which would then be exploited for its localization? How about other protein and mRNA properties? Such analogue signals, which could explain the above observations, would represent a primitive, general mechanism for mRNAs to encode the localization of their cognate proteins beyond more specific mechanisms^21^.

Here we test this hypothesis by examining the general capability of mRNA-coding sequences to encode in their average properties the features of their cognate protein sequences or three-dimensional (3D) structures. In particular, we analyse >500 available amino-acid property scales to characterize different protein sequence properties together with several different physics-based approaches to specifically characterize the hydrophobicity of protein structures. Overall, we focus on a subset of physicochemical properties of mRNA sequences, which depend linearly on their fractional nucleotide composition and the individual quantifiable characteristics (‘weights’) of the four nucleotides. In addition to a number of known mRNA properties such as molecular weight normalized by length, average propensity to be single-stranded or different properties related to mRNA hydrophobicity, we systematically explore a large space of hypothetical scales, thus defining the boundaries of the types of encoding that are at all possible.

## Results

### Encoding protein sequence properties in cognate mRNA

Different physicochemical characteristics of proteins can be estimated computationally from their primary and/or tertiary structures. In the former case, we consider different physicochemical property scales for the 20 canonical amino acids (for example, hydrophobicity, net charge, molecular weight, and so on) and for every protein sequence in the human proteome evaluate the linear average of the property in question.

**Figure 1:**
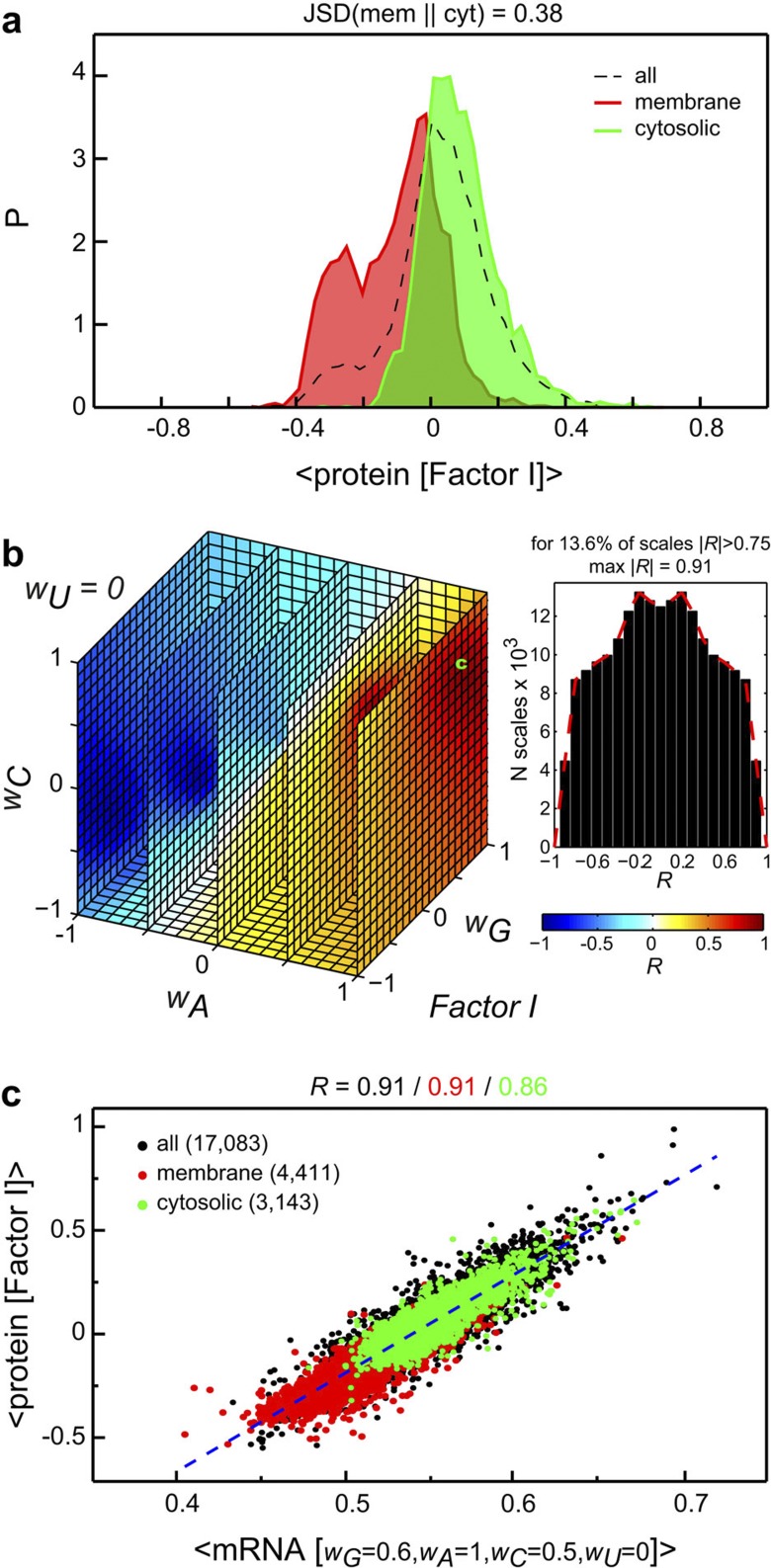
Encoding of average sequence hydrophobicity of proteins in average properties of their cognate mRNA coding sequences. (**a**) Distribution of average protein sequence hydrophobicity as calculated according to the Factor I scale for the entire human proteome (dashed curve), annotated membrane proteins (red filled curve) and annotated cytosolic proteins (green filled curve). (**b**) Distribution of Pearson correlation coefficients *R* (*w*_*G*_, *w*_*A*_, *w*_*C*_, *w*_*U*_) obtained for the human proteome using the Factor I scale (see Methods) and shown as a 3D projection with a fixed value of *w*_*U*_=0 (left) or as a 1D histogram (right). The cube is coloured according to the *R* values as given in the colour legend. The nucleotide scale used for the scatter plot in panel **c** is indicated with ‘c’. (**c**) Scatter plot of average sequence hydrophobicity of human proteins and generalized average mRNA sequence properties calculated using a nucleotide scale that provides the highest value of |*R*|. Annotated membrane and cytosolic proteins are depicted in red and green, respectively, while all other proteins are in black.

We first consider five mutually independent amino-acid property scales, which were obtained by the multivariate statistical analysis of ~500 different amino-acid scales^22^. These five scales can be thought of as the principal components in the space of all amino-acid property scales, each one being representative of a different class of major amino-acid properties: polarity (Factor I scale), secondary structure propensity (Factor II scale), molecular volume (Factor III scale), codon diversity (Factor IV scale) and electrostatic charge (Factor V scale)^22^. Factor I scale in particular reflects amino-acid hydrophobicity and displays a strong correlation with other well-known amino-acid hydrophobicity scales (for example, the Pearson correlation coefficient (*R*) with the widely used Engelman hydrophobicity scale^23^ is 0.93, Supplementary Fig. S1a). The distribution of average sequence hydrophobicities of human proteins according to the Factor I scale (Fig. 1a) is centered at ~0 and shifted slightly in the direction of hydrophilic proteins (positive average Factor I values). Moreover, there is a relatively good separation between the distributions of Factor I average values for all annotated human cytosolic and membrane proteins with a Jensen–Shannon divergence (JSD)^24^ between the two of 0.38 (Fig. 1a).

How well can average physicochemical characteristics of protein sequences, as captured by the five Factor scales, be mirrored in the average sequence properties of cognate mRNA coding regions? To address this question, we exhaustively enumerate all possible generalized property scales for four RNA nucleotides in the range between −1 and 1 using a step of 0.1 (~2 × 10^5^ quadruplets in total). We then compare the average properties of mRNA coding sequences, derived for a given nucleotide scale, against the average properties of their cognate proteins across the entire human proteome. The dynamic range of generalized nucleotide property scales allows for a one-order-of-magnitude difference in values, which we expect to be sufficient for such chemically similar compounds as natural nucleotides. As a measure of matching between protein and mRNA properties, we calculate Pearson correlation coefficients *R* between the average sequence values obtained for a given amino-acid scale and every nucleotide scale from the set of quadruplets. In Fig. 1b we show a projection of *R* (*w*_*G*_, *w*_*A*_, *w*_*C*_, *w*_*U*_) onto a cube with the fixed *w*_*U*_ value of 0 as obtained for the Factor I scale. Remarkably, a large volume of the cube is occupied by high absolute values of *R* with a total of 13.6% of all screened nucleotide scales providing an absolute correlation |*R*| of ≥0.75 over the complete human proteome with the maximum value of max |*R*|=0.91 (Fig. 1b,c). In other words, many different nucleotide property scales allow for a quantitatively accurate, analogue encoding of the average hydrophobicity of protein sequences in the properties of cognate mRNAs, and the very best scales exhibit remarkable encoding potential even among exclusively cytosolic or membrane proteins alone (Fig. 1c). Note that here we only consider the absolute value of the obtained Pearson coefficients given that the sign of nucleotide weights is only a matter of convention.

It is well known that purine-rich codons tend to code for polar amino acids, whereas pyrimidine-rich codons tend to code for hydrophobic amino acids^25^,^26^. Are the above findings just a reflection of this fundamental, well-known feature of the universal genetic code? While qualitatively indeed true, this general property of the genetic code is nonetheless not overly quantitative, with a Pearson *R* between codon PUR content and their cognate amino acids’ Factor I weights of only 0.47 (Supplementary Fig. S2a,b, 22% of variance explained). If one weights the codon PUR content with codon usage bias, the correlation gradually increases to *R*=0.60 (Supplementary Fig. S2a,b, 36% of variance explained). However, only if one considers complete mRNA coding sequences and protein sequences, thus including in a realistic fashion both codon usage bias and realistic amino-acid composition, does the correlation improve to a near-quantitative *R*=0.83 (Supplementary Fig. S2a,b, 69% of variance explained). Finally, weighting the mRNA sequences by the optimized nucleotide scale leads to the ultimate quantitative relationship between protein hydrophobicity and mRNA sequence properties (*R=*0.91, 83% of variance explained; Fig. 1c, Supplementary Fig. S2a,b). Most importantly, such weighting allows one to also consider scales that discriminate between the weights for individual purines (G versus A) and pyrimidines (U versus C). Overall, signal amplification discussed above clearly demonstrates that the connection between protein hydrophobicity and mRNA properties is not just a simple consequence of the genetic code, but rather includes in a complex way also codon usage bias, amino-acid sequence composition and, importantly, nucleotide weights.

**Figure 2:**
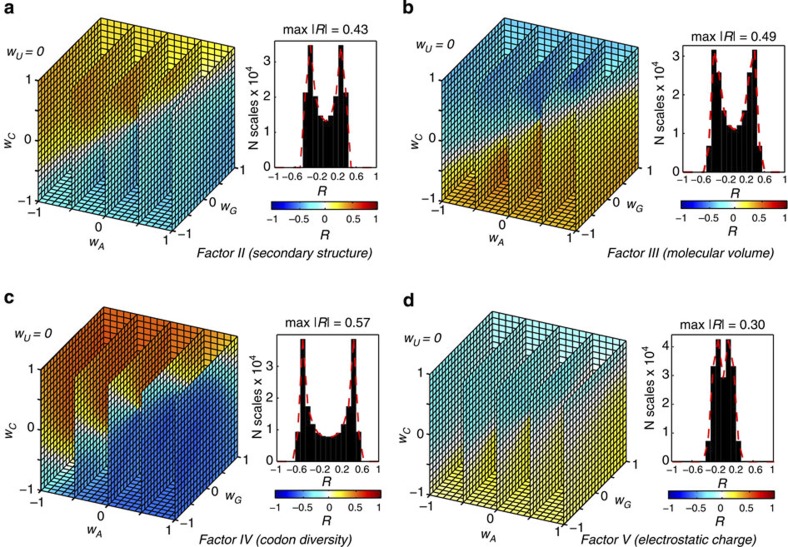
Hydrophobicity-independent average protein sequence properties display a weak connection to cognate mRNA sequences. Distributions of Pearson correlation coefficients *R* (*w*_*G*_, *w*_*A*_, *w*_*C*_, *w*_*U*_) obtained for the human proteome using (**a**) Factor II (secondary structure), (**b**) Factor III (molecular volume), (**c**) Factor IV (codon diversity) and (**d**) Factor V (electrostatic charge) scales, and shown as a 3D projection with a fixed value of *w*_*U*_=0 (*left*) or as a 1D histogram (*right*). Cubes are coloured according to the *R* values as given in the colour legend.

Interestingly, this strong correlation is not seen for protein properties represented by other four Factor scales (Fig. 2). Only for Factor IV, reflecting codon and amino-acid diversity, does one observe moderate correlations (max |*R*|=0.57) between protein and mRNA properties (Fig. 2c). On the other hand, protein electrostatic properties appear to be least encodable in mRNA properties in this sense (Fig. 2d). Finally, none of the examined protein properties except hydrophobicity (Factor I, Fig. 1a) allow for a reasonable discrimination between annotated membrane and cytosolic protein (Supplementary Fig. S3). In other words, average hydrophobicity of protein sequences is not only largely distinct for human proteins with different subcellular localization (membrane or cytosol), but can also be well reflected (that is, encoded) in different sequence-average characteristics of their cognate mRNAs. Very similar results are also observed for representative proteomes from other domains of life (Archea and Bacteria, Supplementary Fig. S4). For Factor I hydrophobicity scale, the values of max |*R*| obtained for *M. jannaschii* (0.88) and *E. coli* (0.85) proteomes are very similar to that of the human proteome, while for most of other Factors the correlations are markedly smaller (especially for *E. coli*, Supplementary Fig. S4).

**Figure 3:**
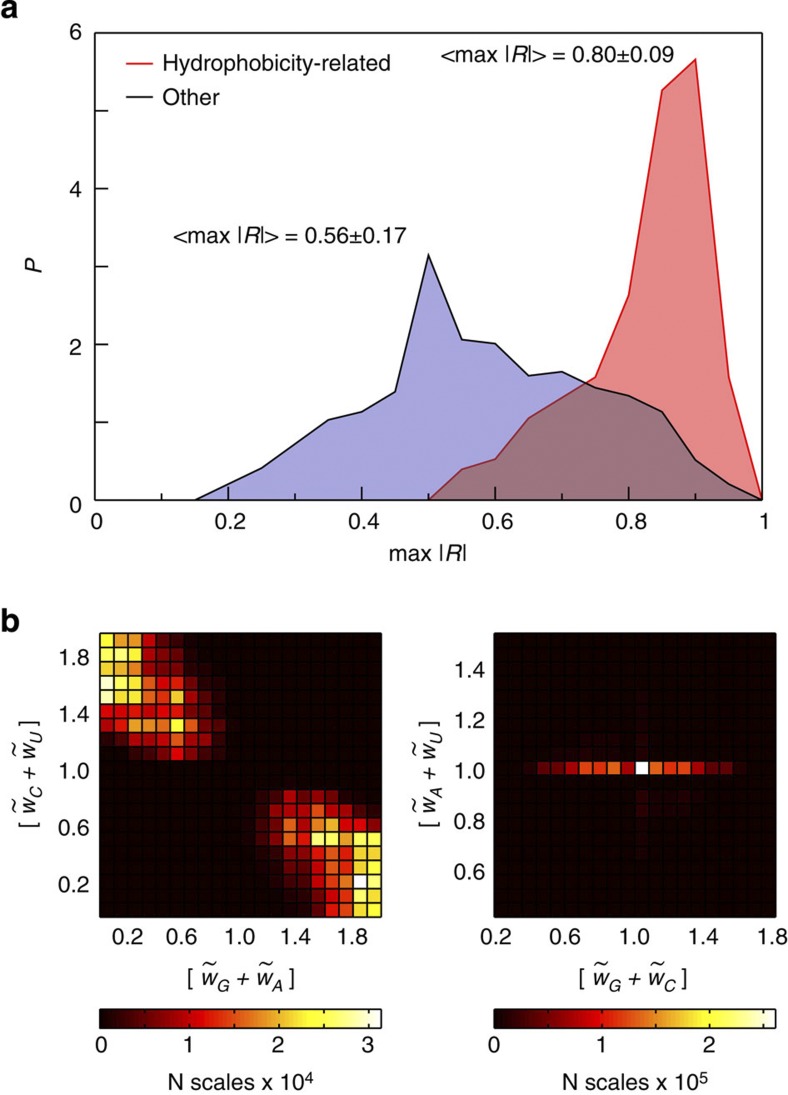
Large-scale analysis of the mRNA encoding potential. (**a**) Distributions of maximum attainable values of |*R*| for 152 hydrophobicity-related (red) and 388 other (blue) amino-acid scales over all generalized nucleotide scales tested for the human proteome. (**b**) For all nucleotide scales that give |*R*|>0.75 for any of 152 hydrophobicity-related scales, we combine the weights for different pairs as indicated after rescaling them between 0 and 1. The heat maps are coloured according to the colour legends given below.

In order to further validate the observed correlation between hydrophobicity and mRNA properties, we perform the same type of calculation of *R* (*w*_*G*_, *w*_*A*_, *w*_*C*_, *w*_*U*_) for the human proteome using 540 different amino-acid property scales (see Supplementary Data 1 for details). As can be seen from the distributions of max |*R*| values obtained independently for 152 hydrophobicity-related and 388 other scales (Fig. 3a), the hydrophobicity-related scales, in contrast to other scales, display very strong matching with generalized average mRNA sequence properties (<max |*R*|>=0.80±0.09, given as mean±s.d.). Overall, optimized nucleotide scales improve correlations for different hydrophobicity scales on average by 0.42 and 0.34 relative to just codon PUR content or codon-usage-bias-weighted codon PUR content, respectively (Supplementary Fig. S2c).

Is there any noticeable pattern among the nucleotide scales that capture protein hydrophobicity well? By rescaling between 0 and 1 all nucleotide scales that provide |*R*|>0.75 for any of the 152 tested hydrophobicity scales (we use ‘~’ to denote rescaled nucleotide scales), we find that they all share a remarkably similar organization. More specifically, all these scales are such that their weights for PUR nucleotides are on average as different as possible from the respective weights for PYR ones. For example, the scale [*w*_*G*_, *w*_*A*_, *w*_*C*_, *w*_*U*_]=[1, 1, 0, 0], corresponding effectively to mRNA PUR content, is one such scale. In Figure 3b, we depict a two-dimensional (2D) density of such scales as a function of the sum of the weights for G and A against the equivalent sum for C and U, which is the same as analysing their respective averages, and show that it is appreciably occupied only in those regions where the two sums are as different as possible (Fig. 3b). An interesting trend is also observed for the 
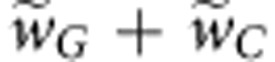
 and 
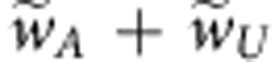
 combination for the scales chosen with the same criterion: while the former sum can be relatively flexible, the latter is almost constant, and the maximum is observed for values of 
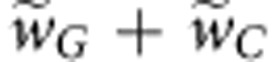
 and 
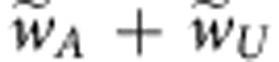
 both equal to 1 (Fig. 3b). Finally, in the case of 
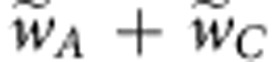
 and 
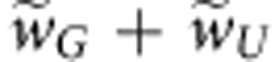
 combinations, the most frequent situation is the one where the summed weights are both equal to 1, but this constraint is significantly more flexible than in previous cases (Supplementary Fig. S5).

### Encoding protein structure hydrophobicity in cognate mRNA

**Figure 4:**
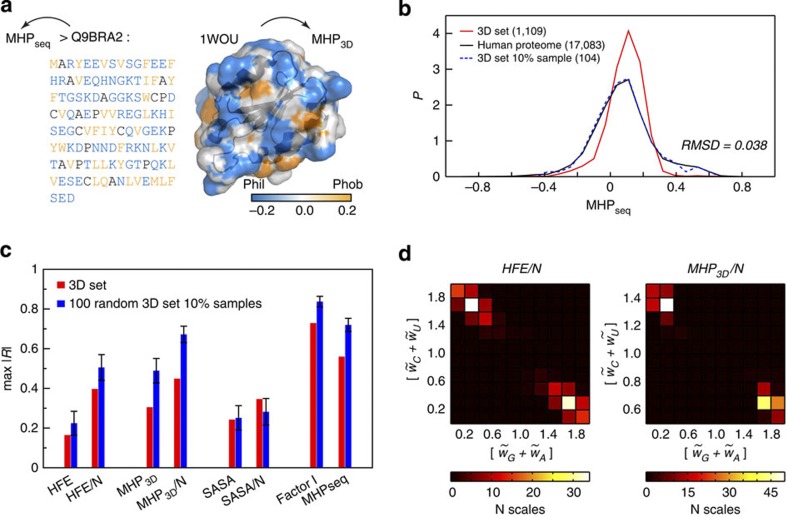
Encoding of protein 3D structure hydrophobicity in generalized characteristics of cognate mRNA sequences. (**a**) Application of MHP approach to the sequence and the 3D structure of a protein. A protein of a representative size from the 3D set human thioredoxin-related protein 14, PDB code: 1WOU, (120 amino acids) is selected as an example and shown in SAS representation. SAS is coloured according to the MHP scale given below. (**b**) Distributions of average sequence hydrophobicities (calculated using an MHP-derived amino-acid scale) for the entire human proteome (black curve), the 3D set (red curve) and a randomly selected sample subset (dashed blue curve). (**c**) Maximum values of |*R|* (max |*R*| and *<*max *|R*|*>* with error bars signifying standard deviations) for different properties of 3D structures and sequences obtained as a result of regular screening of nucleotide scales for the 3D set and 100 random sample subsets (N is the number of protein residues). (**d**) 2D histograms of all rescaled nucleotide scales, which provide the maximum values of |*R*| for MHP_3D_ and HFE of proteins from 100 random subsets, shown as sums of weights for PUR and PYR nucleotides. The heat maps are coloured according to the colour legends given below.

Many proteins require a well-defined spatial structure to properly function in the cell, a common exception being intrinsically disordered proteins^27^. However, in the case of structured proteins, the hydrophobic properties of their folded structures may significantly deviate from those estimated from their sequences only. To address this issue, we also analyse the matching between the average properties of mRNA sequences and the hydrophobicity-related characteristics of 3D structures of their cognate proteins, such as hydration free energy (HFE)^28^ or surface distribution of molecular hydrophobicity potential (MHP)^29^. For these calculations, we use a set of experimentally obtained complete protein structures of various sizes (the most frequent size being 120 residues, Supplementary Fig. S6) in combination with the coding regions of their mRNAs (3D set with 1,109 proteins/mRNAs, see Methods and Supplementary Data 2 for details). The MHP approach utilizes atomic hydrophobic constants derived from water/octanol partitioning experiments on a large number of compounds and allows for hydrophobicity estimation from both the sequence of a protein (based on an MHP-derived amino-acid scale, which is well correlated with the Engelman hydrophobicity scale^23^ with a Pearson *R* of −0.83, Supplementary Fig. S1b) and its spatial structure (according to a formalism, which is analogous to that for calculating the spatial distribution for the electrostatic potential) (Fig. 4a). For every protein in the 3D set, we calculate its HFE, average MHP over its solvent accessible surface (SAS) and sequence MHP (MHP_seq_), and compare these values with the generalized average characteristics of their cognate mRNAs (as based on different nucleotide scales, see above). Significantly, the distribution of the hydrophobic properties of protein sequences from the 3D set is prominently different from that obtained for the whole human proteome (Fig. 4b), which is a consequence of the bias in the Protein Data Bank (PDB) favoring soluble proteins. An important consequence of this is that the dynamic range of structure-based hydrophobicities for the 3D set is narrower than that for the whole proteome. To account for this, we randomly pick ~10% of proteins from the original 3D set whose distribution of MHP-based sequence hydrophobicities closely resembles that for the whole human proteome (root-mean-square deviation of 0.038, Fig. 4b), and perform the respective calculations of HFE and MHP_3D_. Furthermore, we repeat such sub-sampling 100 times and report average values and standard deviations over all of them. As can be seen in Fig. 4c, size-normalized HFE and MHP_3D_ values (HFE/*N* and MHP_3D_/*N* where *N* is the number of protein residues) obtained for such a sub-sample of the 3D set display reasonable correlations with generalized mRNA characteristics with an average max |*R*| of 0.50±0.06 and 0.67±0.04 (both given as mean±s.d.), respectively. Size-normalized hydrophobicities of protein spatial structures match relative mRNA sequence properties better than their absolute values and also exhibit higher correlations for the selected sub-samples than for the entire 3D set. In contrast, no equivalent matching is found for absolute or size-normalized SAS area (SASA or SASA/N, respectively, with max |*R*|<0.35 in all cases). Finally, sequence-derived hydrophobicities (according to Factor I or MHP amino-acid scales) for the analysed proteins display correlations with mRNA sequence properties, which are almost as high as those obtained for the human proteome (Fig. 4c; Supplementary Data 1). Matching is again stronger for the 3D set sub-samples whose distribution mimics the hydrophobic properties of the entire human proteome than for the whole 3D set.

Interestingly, we find that nucleotide weights need to satisfy the same aforementioned constraints (Fig. 3b) to make mRNA able to be representative of protein spatial hydrophobic properties (HFE/*N* and MHP_3D_/*N*). Indeed, according to 2D histograms calculated for the rescaled quadruplets providing max |*R*| for each of the 100 analysed sub-samples of the 3D set, the average values for PUR and PYR weights have to be as different as possible (Fig. 4d).

### Discriminating between membrane and cytosolic proteins

**Figure 5:**
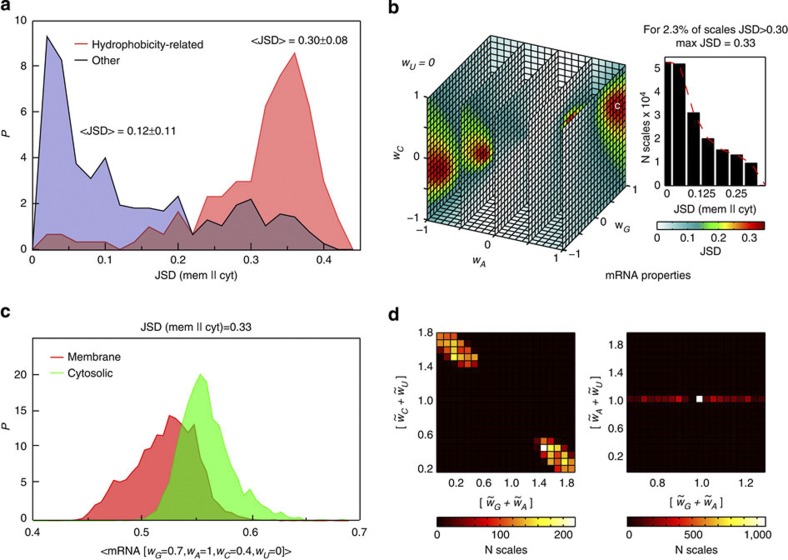
Discriminating between human membrane and cytosolic proteins. (**a**) Distributions of the JSD values obtained from the comparisons of distributions of average sequence properties of human membrane and cytosolic proteins for 152 hydrophobicity-related (red) and 388 other (blue) amino-acid scales. (**b**) *JSD* (*w*_*G*_, *w*_*A*_, *w*_*C*_, *w*_*U*_) distribution obtained from the comparison of human membrane and cytosolic proteins and shown as a projection onto a cube with fixed *w*_*U*_ value of 0 (left) and as a 1D histogram (right). The cube is coloured according to the colour legend given below. Position of the nucleotide scale used for distributions in panel **c** is indicated with ‘c’. (**c**) Distribution of generalized average mRNA sequence properties calculated for the annotated human membrane (red) and cytosolic (green) proteins using a nucleotide scale that provides the highest value of JSD. (**d**) 2D histograms of all rescaled nucleotide scales that provide JSD>0.30, shown as sums of weights for different combinations of nucleotides. The heat maps are coloured according to the colour legends given below.

Which protein sequence properties allow one to discriminate between proteins with different cellular localizations? In the case of cytosolic and membrane proteins, hydrophobicity is arguably the most important such property. Indeed, comparison of generalized property distributions for annotated human cytosolic and membrane proteins calculated for 540 different amino-acid scales clearly shows that hydrophobicity-related scales provide a much more accurate discrimination than other scales, with average JSD values (mem || cyt) of 0.30±0.08 and 0.12±0.11 (both given as mean±s.d.), respectively (Fig. 5a; Supplementary Data 1). If one takes into the account our finding that many generalized average mRNA sequence properties are capable of encoding protein hydrophobicity (see above), we also expect them to be able to discriminate between proteins with different cellular localization. Using a similar framework as above, we calculate four-dimensional (4D) distributions of JSD (*w*_*G*_, *w*_*A*_, *w*_*C*_, *w*_*U*_) values, in each case reporting on the distance between the distributions of average sequence properties of known membrane and cytosolic mRNAs as a function of nucleotide scales (Fig. 5b). This distribution has a different shape as compared with similar distributions of *R* in the case of the Factor I scale (Fig. 1c), and shows that for 2.3% of nucleotide scales, the average sequence characteristics of mRNAs can provide an even more accurate discrimination between membrane and cytosolic proteins (JSD>0.30) than an average hydrophobicity scale. In fact, the best nucleotide scales in this regard exhibit a maximum JSD value of 0.33 (Fig. 5c). For mRNAs to be able to differentiate membrane and cytosolic proteins on average more efficiently than hydrophobicity scales on the side of protein sequences, nucleotide scales have to satisfy similar, but even more rigid constraints compared with those needed to just match protein hydrophobicity, whereby PUR and PYR properties are very distant and the sum of A and U weights exhibits a constant rescaled value of 1 (Fig. 5d). Using the nucleotide scale that optimally discriminates membrane and cytosolic proteins, we show that indeed top and bottom 10% of human mRNAs, as sorted according to their scores, correspond to proteins whose functions clearly link their residence with cytoplasm/nucleus or membrane cellular compartments, respectively (Supplementary Fig. S7).

### Relationship to real nucleotide scales

**Figure 6:**
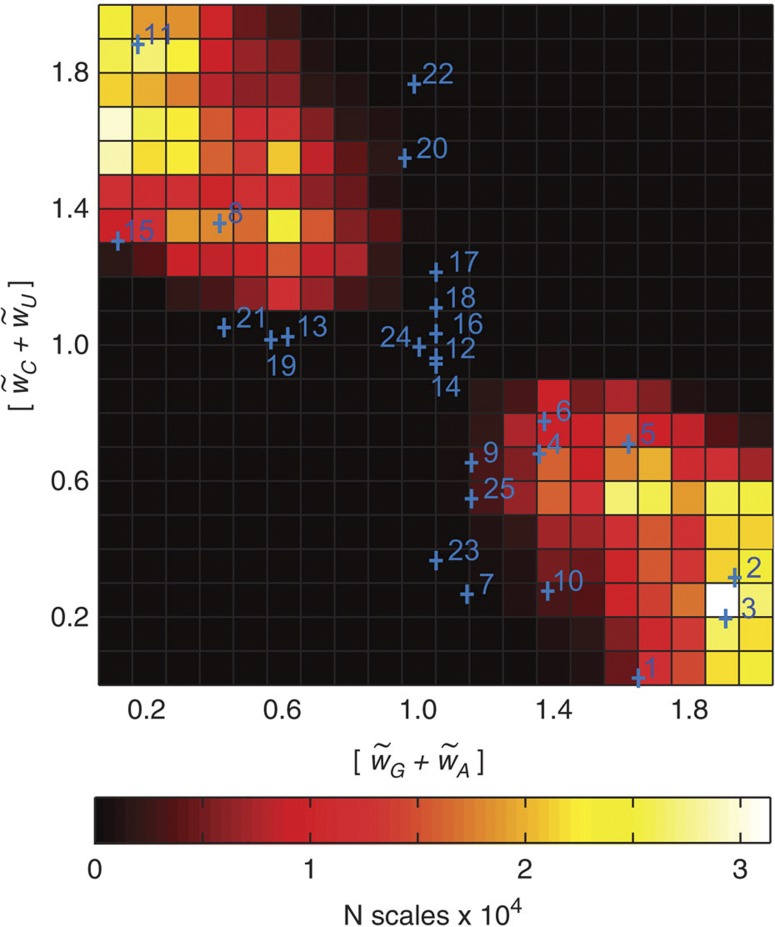
Generalized nucleotide scale constraints and real nucleotide property scales. Real nucleotide scales rescaled between 0 and 1 and overlaid with 2D histograms of summed PUR and PYR weights obtained from calculations of *R* (*w*_*G*_, *w*_*A*_, *w*_*C*_, *w*_*U*_) for mRNA sequence properties and 152 different hydrophobicity scales that give |*R*|>0.75. Positions of summed PUR/PYR values for different real scales are shown with blue crosses and reflect various physicochemical properties of nucleotides (see Methods for full annotation): size/SASA (scales 1–3), knowledge-based contact statistics (scales 4–7), knowledge-based preference of unpaired conformation (scales 8–9) and hydrophobicity-related scores (scales 10–25). The heat map is coloured according to the colour legend given below.

Which real physicochemical properties of nucleotides or nucleobases could be used to encode analogue mRNA signals related to protein hydrophobicity and/or localization to a given cellular environment? To address this question, we have analysed a number of known nucleotide/nucleobase scales (25 in total) rescaled between 0 and 1 and compared them with the previously obtained constraints for generalized nucleotide scales (Fig. 6). These real scales capture different nucleotide or nucleobase properties such as size/SASA (scales 1–3), knowledge-based contact statistics (scales 4–7), knowledge-based preference of being single-stranded (scales 8–9) or various hydrophobicity-related measures (scales 10–25). Size-dependent properties of nucleotides (that is, nucleobases), obviously, match well the requirement that PUR and PYR bases should be clearly distinguishable. For example, molecular weight scores (scale 1), SASA of isolated bases (scale 2), and average contact surface with amino acids (scale 3) occupy the mostly populated regions of 2D histograms for the encodability of protein hydrophobicity (Fig. 6). Interestingly, several scales related to hydrophobicity of nucleotides or nucleobases (for example, scales 10, 11 or 15) also allow for a high degree of encodability as judged by this analysis (Fig. 6), raising an intriguing possibility that hydrophobicity of proteins may actually be encoded in the hydrophobicity of their cognate mRNAs.

## Discussion

We have shown that generalized average characteristics of mRNA-coding regions are able to efficiently reflect the hydrophobicity of cognate proteins at the level of the human proteome, whereas other physicochemical properties of proteins display a much weaker tendency to be predefined by the transcript (Figs 1b, 2 and 3a). Although amino-acid hydrophobicity is known to be related to the composition of its cognate codons by the structure of the genetic code^25^,^26^,^30^,^31^,^32^, here we analyse genome-wide encoding of hydrophobicity of complete proteins in the generalized properties of their cognate mRNA-coding sequences going significantly beyond the simple codon-to-amino acid relationship (Supplementary Fig. S2). However, we should emphasize that the above analysis was performed on mRNA-coding regions only, ignoring the 5′ and 3′-UTRs, which significantly contribute to average sequence properties of mRNAs. Overall, the observed correlations remain very prominent (max |*R*|>0.8) for human mRNAs with UTRs of short-to-moderate length (≤40% of the full transcript length), which accounts for approximately one-third of the analysed set of human full-length transcripts (Supplementary Fig. S8a), whereas they largely vanish for those sequences where UTRs represent >80% of full transcript length (Supplementary Fig. S8a, <10% of all sequences). Although including UTRs for the complete set of human full-length transcripts leads to a drop in the level of correlation for all five Factor scales as compared with the case if one includes coding mRNA regions only, Factor I hydrophobicity still displays a max |*R|* of a sizable 0.67 (Supplementary Fig. S8b). In contrast, none of the Factor scales provide significant correlations for the unspliced versions of transcripts (for example, max |*R|=*0.39 for Factor I scale, Supplementary Fig. S8b). The fact that looking exclusively at coding sequences or sequences with shorter UTRs leads to better correlations may be important in the context of prokaryotic cells whose mRNAs lack long UTRs but still exhibit equally pronounced ability to encode the hydrophobicity of their cognate proteins (Supplementary Fig. S4) as in the case of eukaryotic mRNAs. Finally, we also should mention that our analysis focused only on those net mRNA properties that are linearly dependent on mRNA composition and fixed nucleotide weights. Of course, inclusion of mRNA secondary and tertiary structure information would represent a significant advancement in the present context, but this is currently technically not possible at the complete proteome level.

Importantly, our results suggest that generalized properties of mRNAs coding for proteins with different cellular localization (as an extreme example, here we use cytosolic and membrane proteins, but a similar analysis can be done for more finely defined subgroups) allow for an efficient discrimination between them. In fact, our approach may be used to help rationalize some confounding experimental data on protein localization in the cell. For example, using the nucleotide scale providing the best discrimination between cytosolic and membrane proteins, we estimate the properties of transcripts that code for cytosolic and membrane proteins, but are surprisingly localized in ER and cytoplasm, respectively^33^,^12^; (Supplementary Fig. S9). Interestingly, the mRNA properties of these two subsets of cytosolic and membrane ‘outliers’ are prominently closer to each other than are membrane and cytosolic transcripts on average (Δ_means_=0.016 versus 0.04) (Supplementary Fig. S9). We speculate that in this case anomalous localization of transcripts may be partly driven by their general physicochemical properties as given by our optimized nucleotide scale or its related variants. Furthermore, we observe that optimized nucleotide scales can efficiently separate coding sequences of transcripts with prominent ER or cytosolic localization (top and bottom 10% of the human full-length mRNAs with experimentally verified localization), where particular nucleotide weights providing the best discrimination (with JSD values of 0.35) are very similar to those obtained for all annotated human mRNA-coding sequences of known cytosolic or membrane proteins (Fig. 5c; Supplementary Fig. S10). Such discriminatory ability also corresponds to a reasonable correlation between experimental log_2_(mem/cyt) values and generalized sequence properties of full-length transcripts and their coding sequences with max |*R|* of 0.49 and 0.58, respectively (Supplementary Fig. S10 inset). However, these correlations decrease if one includes transcripts with less prominent localization preferences (that is, smaller absolute values of log_2_(mem/cyt), Supplementary Fig. S10 inset).

Our analysis of real physicochemical property scales for nucleotides or nucleobases suggests that protein hydrophobicity may be successfully encoded in a number of different average mRNA properties including various size-dependent properties, but also hydrophobicity-related properties (Fig. 6). The latter in particular merit further analysis given that one could use them to formulate an exceptionally simple model of encoding (that is, protein hydrophobicity encoded in mRNA hydrophobicity). Moreover, it was previously proposed that anticodon hydrophobicity, especially for the first two anticodon positions, correlates well with the hydrophobicity of cognate amino acids^34^,^35^,^36^. In general, it is reasonable to assume that the average sequence hydrophobicity of a stretch of mRNA is partly related to its nucleotide composition. Chromatographic experiments, in particular, seem to suggest that average nucleotide composition, together with secondary structure preferences, is a key determinant of RNA hydrophobicity^37^,^38^,^39^. However, different hydrophobicity-related scales for nucleobases, nucleosides or nucleotides are significantly less consistent compared with similar scales for amino acids, and greatly depend on the method used for their determination. This, in turn, leads to significant difficulties when trying to assess the possibility that mRNA and cognate protein hydrophobicities may be related. For example, according to the experimental scale of distribution coefficients between water and cyclohexane for nucleobase analogues (scale 14), and most of calculated hydration free energy scales (scales 16, 17 and 19) PUR and PYR have very similar weights, violating the aforementioned constraints (Fig. 6). On the other hand, most of computationally determined partition coefficients in octanol/water or chloroform/water systems (scales 10, 15, 23, 25), and particularly paper chromatography retention times of di-nucleoside monophosphates (scale 11), allow mRNA hydrophobicity estimated in this way to successfully encode the hydrophobicity of a cognate protein (Fig. 6). Importantly, however, depending on the experimental scale used, this encoding may be proportional (that is, hydrophobic mRNAs code for hydrophobic proteins and *vice versa*), but also inversely proportional (that is, hydrophobic mRNAs code for hydrophilic proteins and *vice versa*). For example, the MHP scale (scale 15) belongs to the former group and leads to an |*R*| of 0.60 when used at the whole-human proteome level against the Factor I scale for amino acids, while the scale based on paper chromatography retention times of di-nucleoside monophosphates (scale 11) belongs to the latter group with an equivalent |*R*| of 0.85. Although the former mechanism does have the advantage that it immediately suggests an explanation for translation-independent membrane targeting of membrane proteins’ mRNAs^19^, pronounced inconsistencies in experimental and computational nucleotide or nucleobase hydrophobicity scales weaken any speculation in this direction at present time.

In conclusion, our findings point to a possible existence of a general mechanism of mRNA localization, which may have been operational even in very primitive, ancient cells, but has been tuned by protein machinery in the course of evolution. However, the question remains as to which exact physicochemical properties of mRNAs are utilized, if at all, by the modern cell during sorting and trafficking. We believe our study provides a general framework for addressing this question quantitatively and presents the first view of the key constraints that define the answer to it.

## Methods

### Sequence data sets

The sequences of the complete *Homo sapiens* (human) proteome (17,083 proteins) and coding sequences of their corresponding mRNAs were extracted from UniProtKB database (January 2013 release) and European Nucleotide Archive, respectively. This data set is available as Supplementary Data elsewhere^40^. Data sets for *Methanocaldococcus jannaschii* (1,667 proteins) and *Escherichia coli* (4,149 proteins) were extracted using UniProtKB April 2013 release. Protein as well as RNA sequences with only canonical amino acids or nucleotides were chosen for analysis. Sorting of human proteins into mutually exclusive cytosolic or membrane groups was based on the controlled vocabulary within the ‘Subcellular location’ subsection of each of the UniProtKB entries and using the following criteria: membrane proteins (4,411 in total) are those labelled with any of the ‘Membrane’, ‘Multi-pass membrane protein’, ‘Single-pass membrane protein’, ‘Single-pass type I membrane protein’, ‘Single-pass type II membrane protein’, ‘Single-pass type III membrane protein’ or ‘Single-pass type IV membrane protein’ identifiers, but are not labelled with the ‘Cytoplasm’ identifier, whereas the opposite was used for the cytosolic proteins (3,143 in total). Proteins that did not fall into either category were designated as ‘other’.

### Full-length transcript data set

Full transcripts for the human proteome were obtained from the Ensembl^41^ release 72 (June 2013) by mapping first the UniProt accession numbers (ACs) to Ensembl transcripts, followed by the selection of transcripts with perfectly matched coding sequences to those already present in the starting set (17,083 proteins). In cases where several transcripts were available for a given protein, only the longest transcript was used for the analysis. In this way, the final set of 7,776 proteins and their corresponding full transcripts was generated. In addition, unspliced transcripts for this same set (7,765 in total) were extracted from the Ensembl release 73 (September 2013).

### Data set of protein 3D structures

The set of protein structures was downloaded from the PDB (January 2013 release) using the following criteria: first, X-ray or NMR protein complexes were exclusively monomeric (number of protein entities and the number of chains in the biological assembly fixed at one) with no modified residues; second, total number of entities was fixed at one (that is, structures contained proteins only); third, X-ray structure resolution was better than 2.5 Å. All chains with gaps in the backbone were filtered out using PDB2PQR software^42^ (version 1.6). Only those chains that could be matched to the protein’s canonical UniProtKB sequence were kept for subsequent selection. Furthermore, only the most complete chains were selected, with completeness defined as the ratio of the length of the polypeptide chain in the structure and the length of its canonical UniProtKB sequence. The completeness cutoff for any individual chain was set to >95%. The set was further homology-filtered using the PDB advance search tool for removing similar sequences with the cutoff set at 30% identity. This procedure finally resulted in a set of 1,109 non-redundant monomeric protein structures (3D set, Supplementary Data 2).

### Correlation analysis

Average sequence properties of proteins were estimated using 540 different amino-acid scales (152 hydrophobicity-related and 388 other, hydrophobicity-unrelated scales)^43^ (see Supplementary Data 1 for the details) as follows:


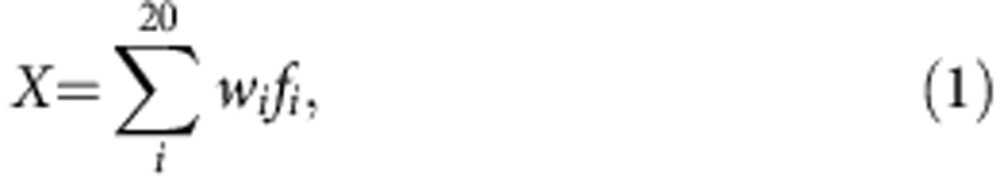


where *X* is sequence property as defined by a given amino acid scale [*w*_1,_…, *w*_20_] and *f*_*i*_ denotes the fraction of residue type *i* in the sequence. Average properties of coding parts of mRNA (*Y*) were calculated similarly to equation 1 using quadruplets of nucleotide weights (*w*_*G*_, *w*_*A*_, *w*_*C*_, *w*_*U*_). Nucleotide scales were screened in a range between −1 and 1 with the step of 0.1 (194,481 quadruplets in total). Pearson correlation coefficients (*R*) between *X* properties of proteins and *Y* (*w*_*G*_, *w*_*A*_, *w*_*C*_, *w*_*U*_) properties of cognate mRNA-coding sequences were calculated for each amino-acid scale, which results in 4D distributions of *R* (*w*_*G*_, *w*_*A*_, *w*_*C*_, *w*_*U*_) in each case. These distributions were visualized as 3D projections onto the (*w*_*G*_, *w*_*A*_, *w*_*C*_) cube with a fixed *w*_*U*_ weight of 0. All quadruplets providing |R|*>*0.75 for hydrophobicity-related scales were rescaled between 0 and 1 ([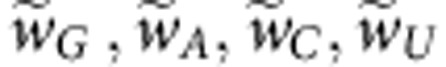]) and represented as 2D histograms of summed weights for all possible nucleotide pairs (for example, 
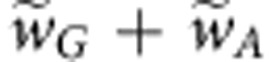
 and 
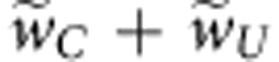
 for PUR and PYR, respectively). All the calculations and visualizations were done using MATLAB (R2009a).

### Hydrophobicity of protein 3D structures

Hydration free energy (HFE) of energy-minimized structures from the 3D set was calculated using generalized Born/surface area (GB/SA) methodology^28^ with OPLSaa force field parameters^44^. Energy minimization and HFE calculation was performed using molecular modelling package TINKER^45^ (version 5.1).

As a measure of hydrophobicity of proteins from the 3D set, a distribution of MHP values^29^ mapped onto protein SAS was also used. The formalism of MHP is based on empirical atomic hydrophobicity constants (that is, hydrophobicity ‘charges’) derived from partition coefficients, Log *P*, of various compounds between polar and apolar media (for example, water/n-octanol). In analogy with the electrostatic Coulomb potential, MHP is constructed to have exponential distance dependence. Thus, contribution of *N* atoms to MHP at point *i* can be estimated as follows:


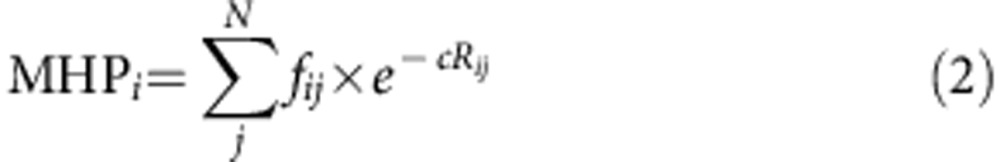


where *f*_*j*_ is atomic hydrophobicity constant of atom *j*, *R*_*ij*_ is the distance between atom *j* and point *i*, and *c* is a decay constant (here we used *c* of 0.5 Å (ref. 46)). SAS calculation and mapping of MHP onto protein surface in each of its points were performed using PLATINUM software^47^. Further analysis of MHP data was carried out using utilities written especially for this. The MHP values were expressed in octanol/water Log *P* values (base-10 logarithm of octanol/water partition coefficients). The sum of MHP values on protein surface (MHP_3D_) was used as a measure of protein 3D-structure hydrophobicity.

The thus-obtained absolute and relative HFE and MHP_3D_ values (normalized by the number of protein residues) were used in calculations of *R* (*w*_*G*_, *w*_*A*_, *w*_*C*_, *w*_*U*_) as described in the previous section. Average sequence properties of proteins in the 3D set were calculated using MHP-derived (MHP_seq_) and Factor I (ref. 22) amino-acid scales according to Equation 1. These calculations were also performed for 100 randomly selected subsets of the 3D set, whose distributions of MHP_seq_ fit best to the one of the entire human proteome. These representative subsets were obtained by selecting proteins with frequencies that resemble frequencies of MHP_seq_ values obtained for the human proteome (bin size 0.07). As a result, the size of every random subset was 104 proteins and the calculated MHP_seq_ distributions displayed a correlation of 0.99 to the human MHP_seq_ distribution and root-mean-square deviation of 0.038 from the reference. All quadruplets providing maximum absolute *R* (*w*_*G*_, *w*_*A*_, *w*_*C*_, *w*_*U*_) for each of 100 random samples were rescaled between 0 and 1 and represented as 2D histograms of summed weights for all possible nucleotide pairs. All of the above calculations and visualizations were done using MATLAB (R2009a).

### Estimation of discriminatory power

Distances between distributions of protein or mRNA sequence properties calculated separately for human membrane and cytosolic proteins were estimated according to the JSD formalism^24^:


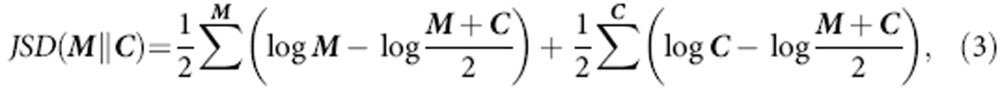


where ***M*** is a distribution of sequence properties for membrane proteins and ***C*** is a distribution for cytosolic proteins, and the logarithm is base 2. For every distribution, a standard binning scheme was used, where 50 bins between minimum and maximum values of the property in question were applied. JSD (mem || cyt) values for protein sequence properties were calculated for all 540 amino-acid scales. For mRNA sequences of human membrane and cytosolic proteins, a 4D distribution of JSD (*w*_*G*_, *w*_*A*_, *w*_*C*_, *w*_*U*_) was calculated using a screening procedure as described above. All quadruplets providing JSD (*w*_*G*_, *w*_*A*_, *w*_*C*_, *w*_*U*_)>0.30 were rescaled between 0 and 1 and represented as 2D histograms of summed weights for all possible nucleotide pairs. All of the above calculations were performed using utilities specially written for this purpose. Visualization was done in MATLAB (R2009a).

### Gene ontology (GO) analysis

DAVID Bioinformatics Resources tool^48^ (version 6.7) was used for the functional enrichment analysis of the top and bottom 10% mRNA scores generated with the nucleotide scale that discriminates the best between cytosolic and membrane proteins. UniProt ACs were used as input and only the biological processes and molecular functions belonging to the GO FAT collection of GO terms were considered. Final heat maps report the obtained EASE scores for the 10 most significant and non-redundant GO terms.

### Analysis of experimental mRNA partitioning data

The mRNA partitioning data for the human myelogenous leukaemia K-562 cell line was originally reported in the study by Diehn *et al.*^33^, while for the purposes of this analysis a filtered and sorted version of this data as reported in the study by Chen *et al.*^12^ was used. We extracted as described above a set of transcripts with annotated localization, known membrane/cytosol partitioning values and available full-length mRNA sequences (2,741 mRNAs). In addition, mRNAs encoding cytosolic/nuclear or endomembrane proteins were sorted by their corresponding log_2_(mem/cyt) ratios, and the top 20 outliers for each cohort (that is, those with highest positive log_2_(mem/cyt) values among cytosolic/nuclear, and those with highest negative log_2_(mem/cyt) values among membrane proteins) were selected for further analysis.

### Fitting of real nucleotide/nucleobase scales

Twenty five different real nucleotide/nucleobase scales were rescaled between 0 and 1 and overlaid with 2D histograms of summed PUR and PYR scores obtained from calculations of *R* (*w*_*G*_, *w*_*A*_, *w*_*C*_, *w*_*U*_) for hydrophobicity scales. Among them are: nucleotide weights (scale 1), isolated base SASA (scale 2), average base contact surface with protein residues and contact preferences of bases obtained from analysis of protein–RNA interfaces in a large set of PDB structures^40^ (scales 3 and 4, respectively), knowledge-based fraction of contacts on protein–RNA interfaces^49^,^50^ (scales 5 and 6, respectively), knowledge-based fraction of contacts between nucleobases and amino-acid side chains at protein–RNA interfaces and log-odds preference of unpaired conformations for protein–RNA interfaces versus protein-free RNA regions^51^ (scales 7 and 8, respectively), knowledge-based fraction of unpaired nucleotides^52^ (scale 9), extrapolated octanol–water partition coefficients^53^ (scale 10), paper chromatography retention times for di-nucleoside monophosphates^34^ (scale 11), water-affinities in chloroform and 2-butanol^54^ (scales 12 and 13, respectively), partition-free energies in the cyclohexane–water system^55^ (scale 14), MHP-derived hydrophobicities (scale 15), calculated solvation-free energies in water^56^,^57^,^58^,^59^ (scales 16–19, respectively), calculated solvation-free energies in chloroform^58^,^59^,^60^ (scales 20–22, respectively), calculated partition coefficients in chloroform–water system^58^,^59^,^60^ (scales 23–25, respectively). If not indicated otherwise, all of the above scales were originally derived for nucleobases.

## Author contributions

A.A.P. performed computational research with help of M.H. All authors participated in the designing of the research, analysing the data and writing the paper.

## Additional information

**How to cite this article:** Polyansky, A. A. *et al.* Analogue encoding of physicochemical properties of proteins in their cognate messenger RNAs. *Nat. Commun.* 4:2784 doi: 10.1038/ncomms3784 (2013).

## Supplementary Material

Supplementary Figures Supplementary Figures S1-S10

Supplementary Data 1 JSD and max |R| values for 540 amino-acid scales obtained as a result of grid-based scanning of nucleotide weights for the human proteome

Supplementary Data 2 The 3D set of protein spatial structures
