# Supplementary material for: Analogue encoding of physicochemical properties of proteins in their cognate messenger RNAs
Source: Nat Commun. 2013 Nov 20;4:2784. doi: 10.1038/ncomms3784 (PMC3868254; doi:10.1038/ncomms3784)
Supplement: Supplementary Figures — S1-S10 [file ncomms3784-s1.pdf]

Supplementary Information

Analogue encoding of physicochemical properties of proteins in their cognate messenger RNAs

Anton A. Polyansky, Mario Hlevnjak & Bojan Zagrovic*

Department of Structural and Computational Biology, Max F. Perutz Laboratories, University of Vienna, Campus Vienna Biocenter 5, A-1030 Vienna, Austria

*corresponding author: bojan.zagrovic@univie.ac.at

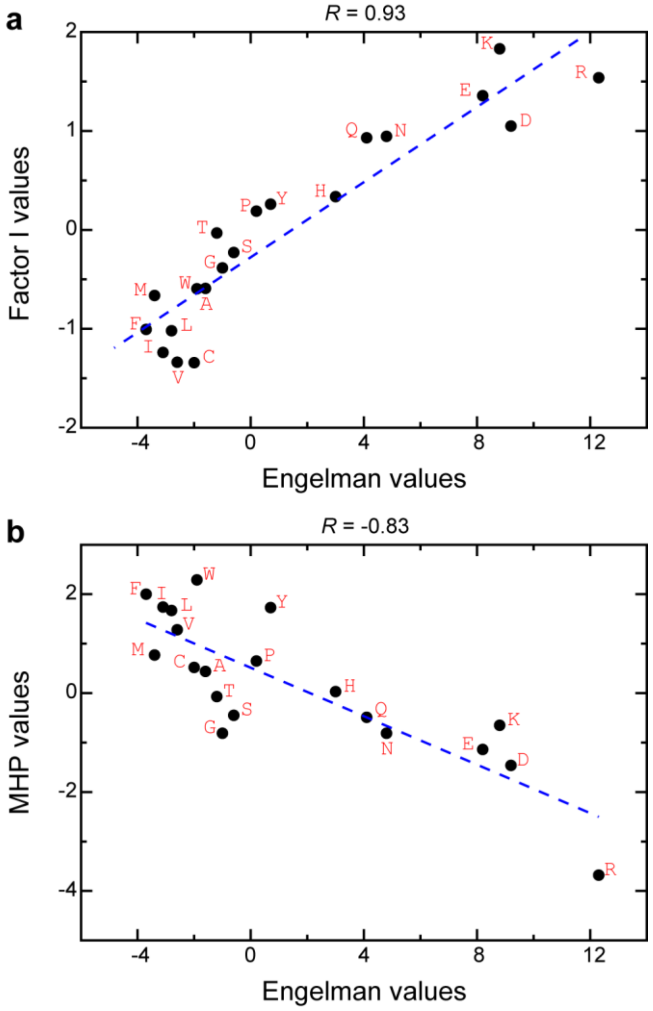

Supplementary Figure S1 | Validation of Factor I and MHP scales. Correlation of (**a**) Factor I and (**b**) MHP amino-acid scales with the Engelman hydrophobicity scale [23].

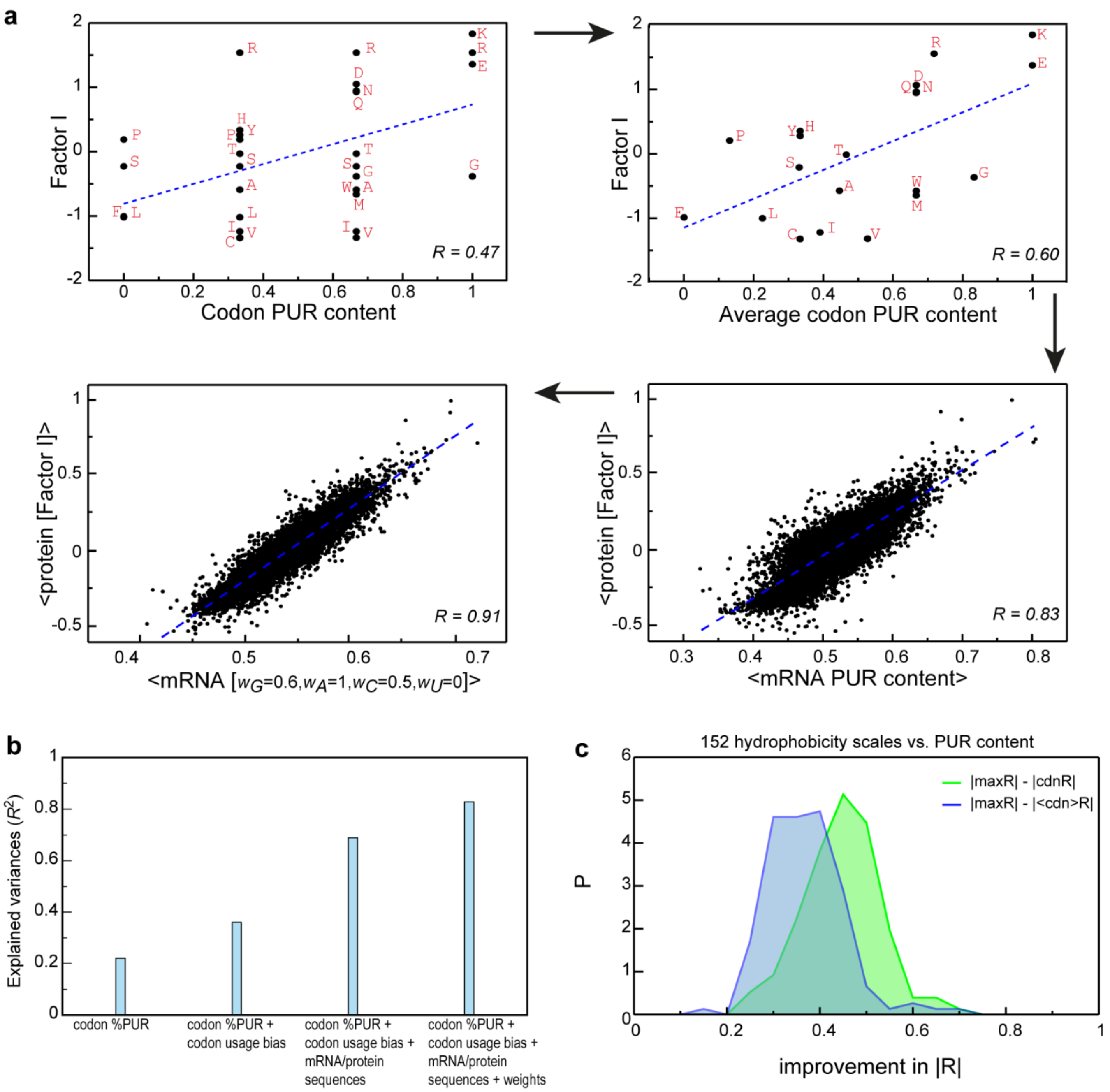

Supplementary Figure S2 | Contributions to mRNA encoding potential. Correlation between codon PUR content and amino-acid hydrophobicity as well as between mRNA properties and protein hydrophobicity. (**a**) In a clockwise order: correlation between Factor I of amino acids and the PUR content of their individual codons; correlation between Factor I of amino acids and the average PUR content of their codons in mRNAs of the entire human proteome; correlation between average sequence hydrophobicity of human proteins and PUR content of their cognate mRNAs; correlation between average sequence hydrophobicity of human proteins and generalized average mRNA sequence properties calculated using the nucleotide scale which provides the highest value of *R*. (**b**) Fraction of total variance captured by the linear correlation in different cases. (**c**) Distribution of differences between proteome-scale max $|R|$ values (improvements in $|R|$), obtained by weighted mRNA/protein comparison for 152 different amino-acid hydrophobicity scales, and correlation coefficients, obtained by comparing each of these scales against the PUR content of respective codons weighted by codon-usage bias (blue) or not (green).

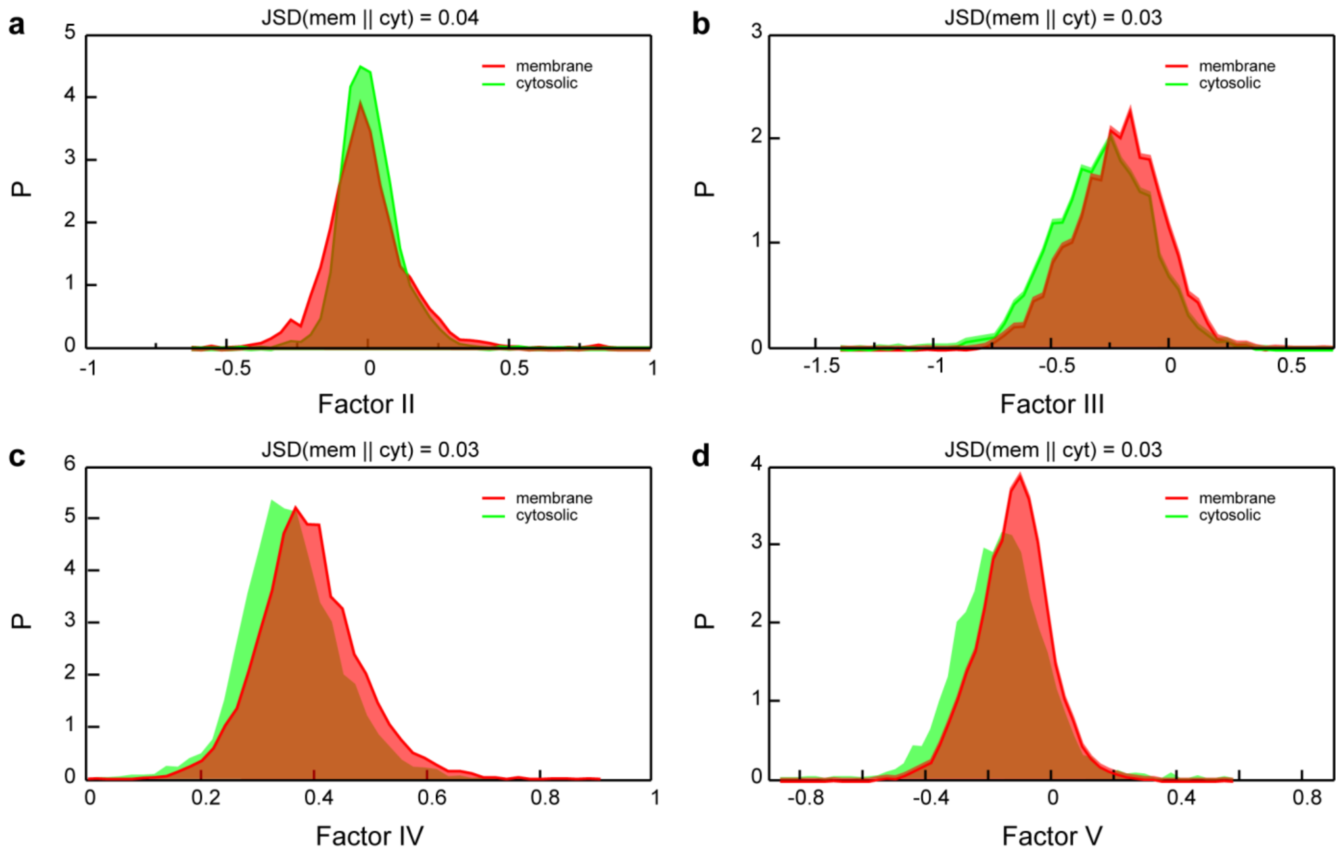

Supplementary Figure S3 | Discrimination provided by Factors II-V. Distribution of protein sequence properties calculated according to Factor II, III, IV and V amino-acid scales for the annotated human membrane (red) and cytosolic (green) proteins.

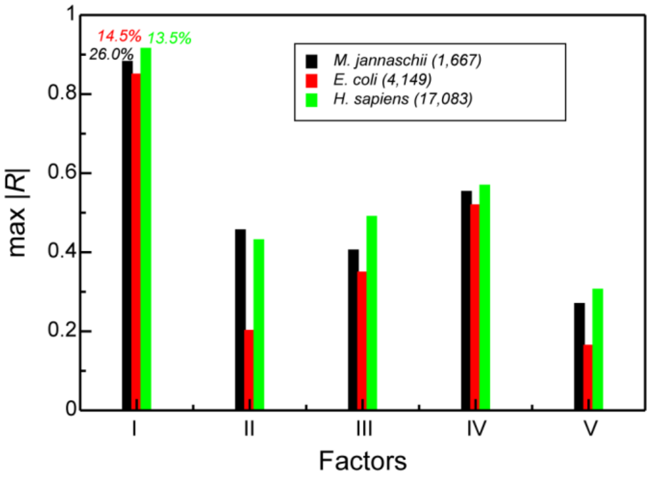

Supplementary Figure S4 | Correlations in different domains of life. Values of max |*R*| for different Factor scales and proteomes of an archeon (*Methanocaldococcus jannaschii*), a bacterium (*Escherichia coli*) and a eukaryote (*Homo sapiens*), with proteome sizes given in the legend. Fraction of scales providing |*R*| ≥ 0.75 are given for Factor I above the graphs.

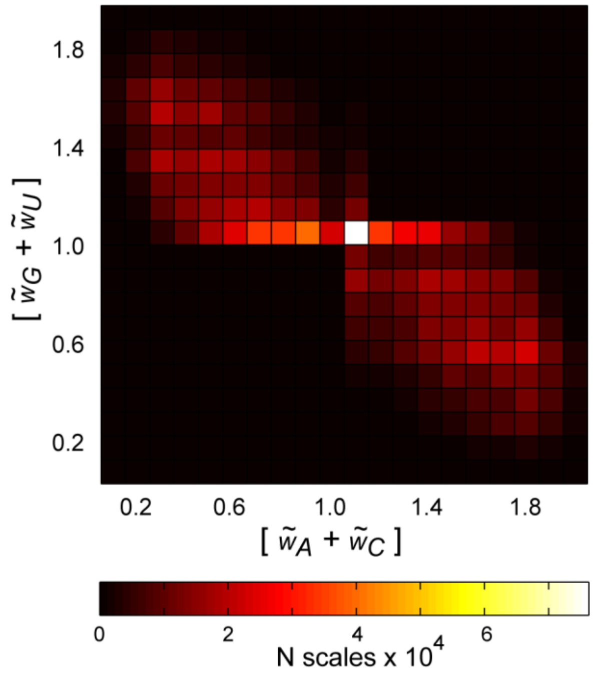

Supplementary Figure S5 | Additional constraints for nucleotide weights. 2D histograms of all rescaled nucleotide scales which provide $|R| > 0.75$ for 152 hydrophobicity-related amino-acid scales, shown as sums of weights for A and C on the x-axis and G and U on the y-axis. The heat map is colored according to color legend given below the panel.

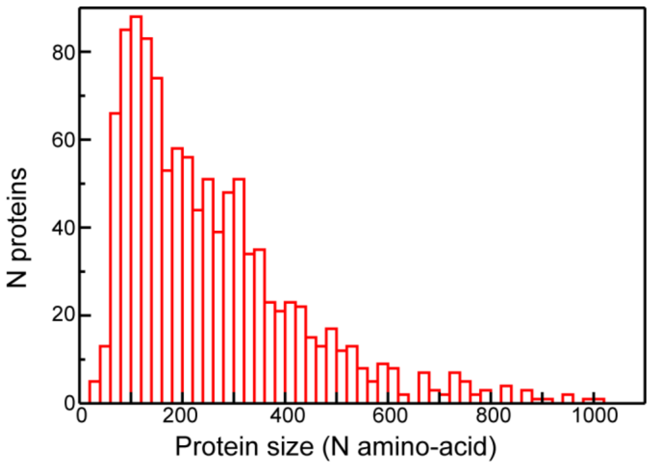

Supplementary Figure S6 | 3D set characterization. Distribution of protein sizes in the 3D set.

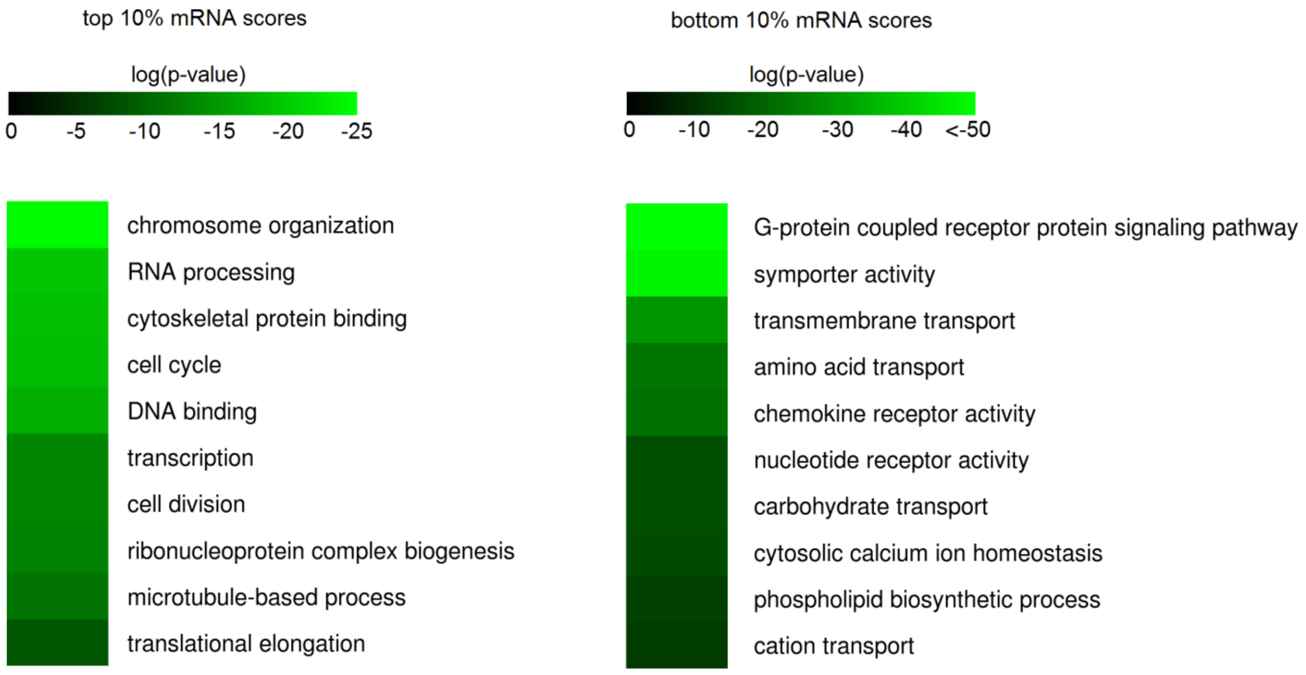

Supplementary Figure S7 | Biological illustration of optimal discrimination. Results of Gene Ontology (GO) analysis performed for top (*left*) and bottom (*right*) 10% of human mRNAs (coding sequence only) sorted by their average sequence properties as calculated according to the nucleotide scale providing the best discrimination between cytosolic and membrane proteins (Figure 5c). As illustrated here, mRNA properties can successfully be used to differentiate between cytosolic/nuclear and membrane proteins as indicated by their specific functions.

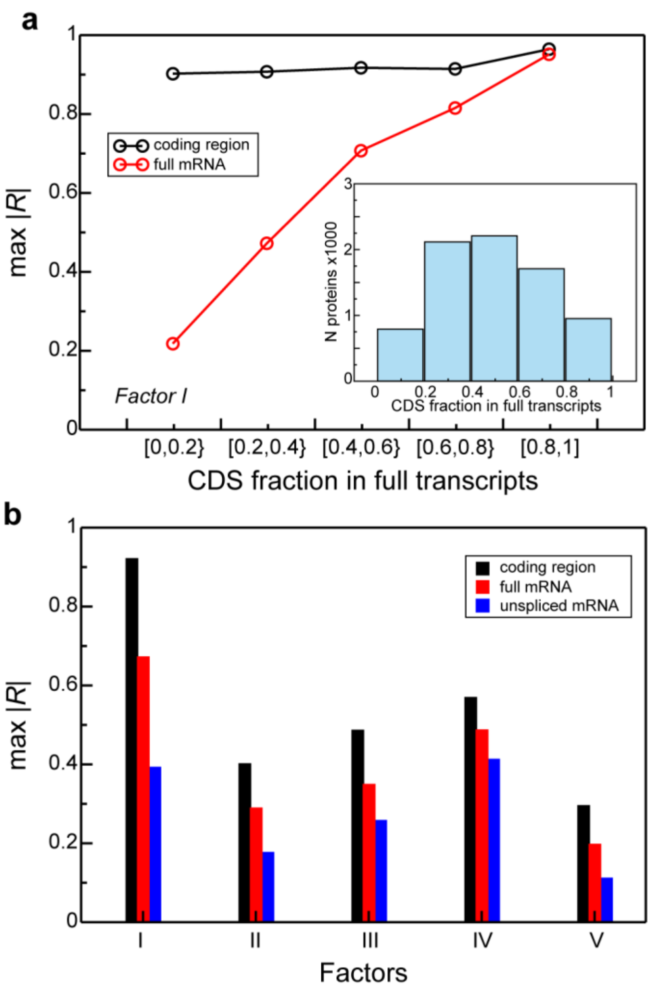

Supplementary Figure S8 | Effect of UTRs and splicing on correlations. (**a**) Dependence between the relative coding-sequence length in full mRNAs and Factor I max |*R*|. *Inset:* Distribution of the number of mRNAs having different relative coding sequence lengths. (**b**) max |*R*| for different Factors for all full-length human transcripts available (a total of 7,776 sequences) as well as their coding and unspliced sequences.

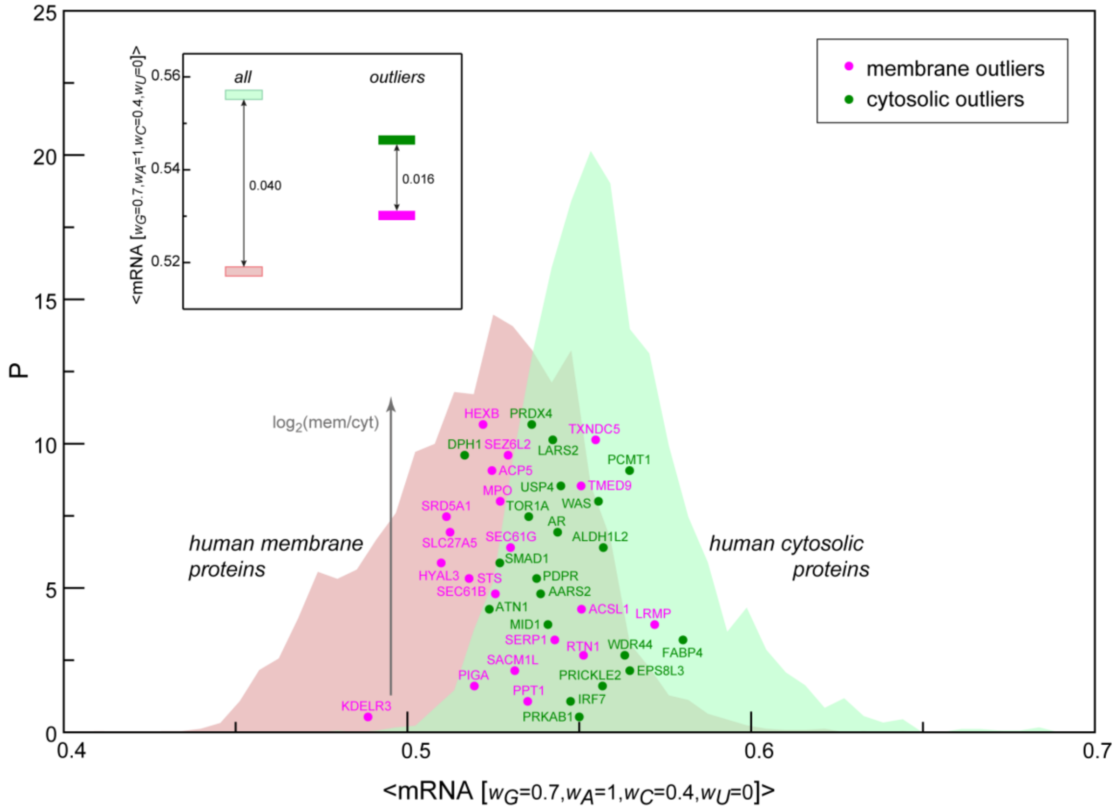

Supplementary Figure S9 | Localization outliers. Properties of coding sequences of human mRNAs of cytosolic (dark green dots) and membrane (dark magenta dots) proteins which display anomalous localization to ER and cytoplasm, respectively [12,33]. Complete distributions of all human membrane (pale red) and cytosolic (pale green) transcripts are shown in the background. Positions of "outlier" transcripts are given with respect to their mRNA score (x-axis) and relative distribution coefficients between membrane and soluble fractions (log_2(mem/cyt) [12,33], vertical). *Inset*: difference between the average mRNA sequence properties of all human membrane and cytosolic mRNAs as compared to the difference between the average mRNA sequence property of "outlier" proteins.

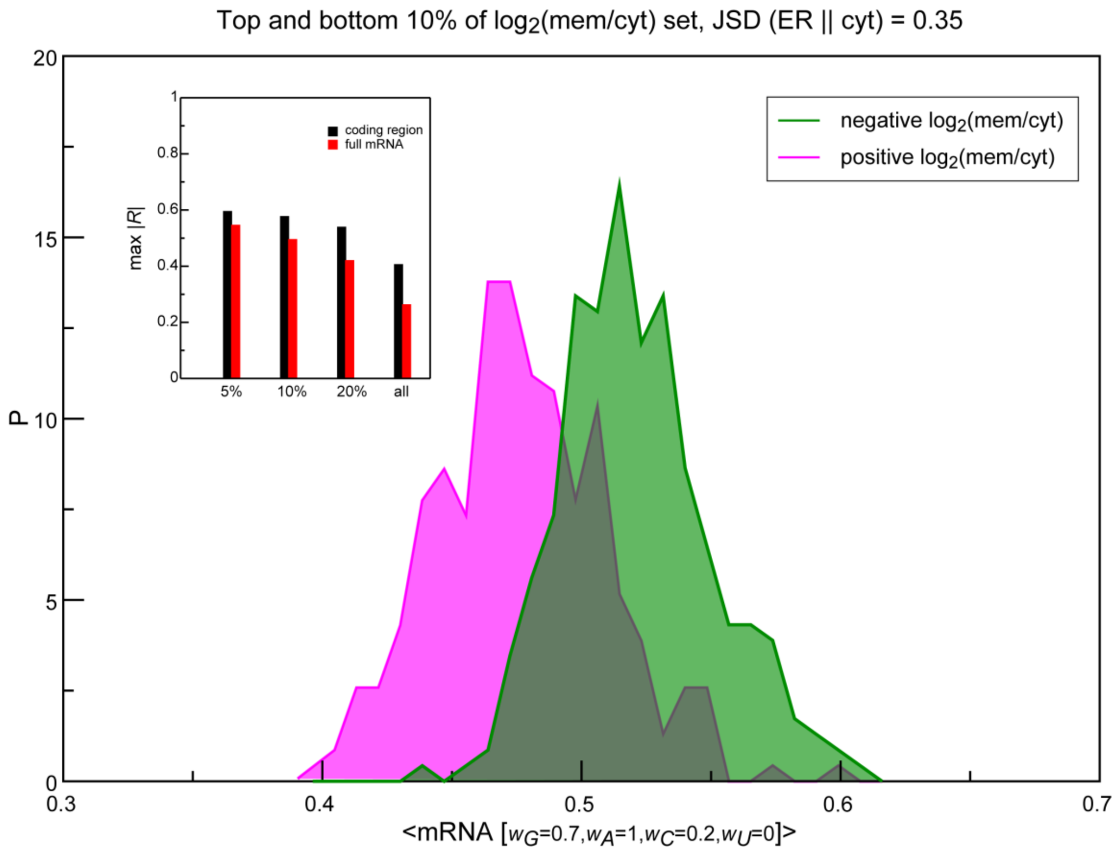

Supplementary Figure S10 | Analysis of mRNAs with known localization. Distribution of generalized average mRNA sequence properties calculated for human transcripts known to localize to the ER (magenta) or in the cytoplasm (dark green). The values are calculated for the 10% of transcripts with extremely positive (ER-localized) or negative (cytoplasm-localized) $\log_2$(mem/cyt) values using a nucleotide scale which provides the highest value of JSD in this case. *Inset:* max |*R*| between mRNA sequence properties and experimental $\log_2$(mem/cyt) values for 5%, 10%, 20% extremes and the complete sets of full-length human transcripts (a total of 2,741 sequences) and their coding sequences.
